# The Influence of Parental Control and Parent-Child Relational Qualities on Adolescent Internet Addiction: A 3-Year Longitudinal Study in Hong Kong

**DOI:** 10.3389/fpsyg.2018.00642

**Published:** 2018-05-01

**Authors:** Daniel T. L. Shek, Xiaoqin Zhu, Cecilia M. S. Ma

**Affiliations:** ^1^Department of Applied Social Sciences, The Hong Kong Polytechnic University, Hong Kong, China; ^2^Centre for Innovation Programmes for Adolescents and Families, The Hong Kong Polytechnic University, Hong Kong, China; ^3^Department of Social Work, East China Normal University, Shanghai, China; ^4^Kiang Wu Nursing College of Macau, Macau, China; ^5^Hong Kong Institute of Service Leadership and Management Limited, Hong Kong, China; ^6^Division of Adolescent Medicine, Kentucky Children's Hospital, University of Kentucky, Lexington, KY, United States

**Keywords:** internet addiction, longitudinal study, family, Hong Kong, individual growth curve

## Abstract

This study investigated how parental behavioral control, parental psychological control, and parent-child relational qualities predicted the initial level and rate of change in adolescent internet addiction (IA) across the junior high school years. The study also investigated the concurrent and longitudinal effects of different parenting factors on adolescent IA. Starting from the 2009/2010 academic year, 3,328 Grade 7 students (*M*_*age*_ = 12.59 ± 0.74 years) from 28 randomly selected secondary schools in Hong Kong responded on a yearly basis to a questionnaire measuring multiple constructs including socio-demographic characteristics, perceived parenting characteristics, and IA. Individual growth curve (IGC) analyses showed that adolescent IA slightly decreased during junior high school years. While behavioral control of both parents was negatively related to the initial level of adolescent IA, only paternal behavioral control showed a significant positive relationship with the rate of linear change in IA, suggesting that higher paternal behavioral control predicted a slower decrease in IA. In addition, fathers' and mothers' psychological control was positively associated with the initial level of adolescent IA, but increase in maternal psychological control predicted a faster drop in IA. Finally, parent-child relational qualities negatively and positively predicted the initial level and the rate of change in IA, respectively. When all parenting factors were considered simultaneously, multiple regression analyses revealed that paternal behavioral control and psychological control as well as maternal psychological control and mother-child relational quality were significant concurrent predictors of adolescent IA at Wave 2 and Wave 3. Regarding the longitudinal predicting effects, paternal psychological control and mother-child relational quality at Wave 1 were the two most robust predictors of later adolescent IA at Wave 2 and Wave 3. The above findings underscore the importance of the parent-child subsystem qualities in influencing adolescent IA in the junior high school years. In particular, these findings shed light on the different impacts of fathering and mothering which are neglected in the scientific literature. While the findings based on the levels of IA are consistent with the existing theoretical models, findings on the rate of change are novel.

## Introduction

With the rapid development of information technology, approximately 48% of the world population are using the Internet and over four-fifths of the world's youth population are online (International Telecommunication Union, 2017). Although the dramatic increasing usage of the Internet facilitates many aspects of people's lives, it brings about a growing trend of Internet addiction (IA). IA is conceived as “unregulated use of the Internet which leads to the development of symptoms such as cognitive and behavioral preoccupation with the Internet” (Shek et al., 2015, p. 293), which would lead to physical and psyhcological problems among adolescents (Young, 1998; Kim et al., 2006; Yen et al., 2007; Lin et al., 2013; Cheng and Li, 2014).

To tackle and prevent the problem of adolescent IA, there is a need to understand the development of IA (Shi et al., 2017). A considerable number of researchers came to realize that parents, as the primary source of social influence, may play a vital role in the emergence of adolescent IA (Shi et al., 2017). For instance, parental monitoring, and parental bonding were found to be negatively linked with children's IA (Siomos et al., 2012; Ding et al., 2017). However, most studies did not simultaneously investigate different processes of parent-child subsystem qualities such as behavioral control, psychological control, and parent-child relational qualities.

Behavioral control refers to parents' use of explicit control strategies, such as monitoring children's activities and whereabouts as well as using rules and restrictions to manage children's behaviors (Shek, 2005). In contrast, psychological control focuses on implicitly manipulating children's behaviors through regulating their emotions, thoughts, and feelings (Smetana and Daddis, 2002). Tactics of psychological control include personal attacks, inducing guilt, authority assertion, and love withdrawal. Scholars concluded that while “behavioral control communicates that a behavior is unacceptable, psychological control communicates that the adolescent's thoughts, emotions, feelings, and/or even the adolescent are unacceptable” (Rogers et al., 2003, p. 350). From the existing scientific literature, while parental behavioral control is positively linked to adolescent developmental outcomes, psychological control impairs adolescent development (Barber et al., 2005; Wang et al., 2007; Bleakley et al., 2016).

It is argued that behavioral and psychological control would exert distinct effects on adolescent IA as well. If parents firmly regulate the child's online activities (e.g., when and how the children are allowed to use the Internet) in a warm and consistent manner, the children would be less likely to get addicted to the Internet. In contrast, parental psychological control negatively affects their children's sense of autonomy, identity, and competence (Barber, 2002), which may result in children's excessive Internet use as a dysfunctional way of satisfying the psychological needs (Yu et al., 2012). Empirical evidence has supported these arguments. For instance, the negative association between parents' behavioral control (e.g., parental monitoring) and adolescent IA was repeatedly reported in studies conducted in different communities (Lin et al., 2009; Bleakley et al., 2016; Ding et al., 2017). On the other hand, psychological control and its related factors such as overintrusive parenting and authoritarian parenting were positively related to adolescent IA (Giles and Price, 2008; Huang et al., 2010; Cheung et al., 2015). Despite these accumulated empirical evidences, there are several research gaps in the literature. First, most of the studies did not simultaneously examine behavioral control and psychological control and compare their different influences.

Second, while some scholars suggested that a positive relationship between parents and children is “the fundamental platform” that gives rise to parental control (Crouter and Head, 2002, p. 472), the existing studies have seldom simultaneously investigated parental control and relational qualities of parent-child dyads in the field of IA. Parent-child relational quality refers to the nature of parent-child relationship, such as trust between adolescents and their parents, adolescents' willingness to communicate with their parents and their satisfaction with the parents' control (Shek, 2005). On one hand, there would be a close linkage between relational qualities and parental control. For instance, adolescents' willingness to communicate with their parents would enhance behavioral control (e.g., awareness of the child's daily life) due to children's voluntary self-disclosure (Shek and Law, 2014). On the other hand, adolescents' addicted Internet use can at least be partially attributed to an attempt to cope with unfavorable family relationships (Bleakley et al., 2016). In contrast, if the children have good relationships with their parents, one can anticipate children's higher willingness to be socialized in a way in line with their parents' expectation.

The third research gap is that few studies have distinguished between paternal and maternal effects and the limited existing research findings are not conclusive. In some studies, researchers simply asked adolescents to indicate their perceptions of their parents and investigated the relationship between the aggregated parenting and adolescent IA (Li et al., 2013; Bleakley et al., 2016; Ding et al., 2017). It is possible that mothering is more important than fathering in influencing children's behavior including IA (Burk and Laursen, 2010). For example, in a study involving 178 secondary school students in Australia (Giles and Price, 2008), it was observed that mothers' psychological control served as a positive predictor of children's problematic computer use, whereas fathers' psychological control did not. The authors argued that mothers may have greater psychologically controlling behavior than fathers and thus maternal control resulted in the child's greater problematic computer use as a means of escaping from such control. In a more recent study which utilized a sample of 5,122 Chinese adolescents, (Xu et al., 2014) found that mother-child relationship presented a stronger association with adolescent IA than did father-child relationship. The authors argued that fathers in Chinese culture might be detached from the child's activities. However, some studies highlighted the unique value of fathering in children's development (Shek, 1999a,b; Parke, 2002). For instance, Pleck and Masciadrelli (2004) reviewed studies on fathers' involvement and identified a positive association between fathers' involvement and children's favorable development. In supporting the unique role played by fathers, Lansford et al. (2014) found that it was fathers', but not mothers' psychological control that explained unique variance in the child's developmental problems and only paternal knowledge (i.e., one aspect of behavioral control) showed a unique predicting effect on boys' externalizing problems.

The fourth research gap is that most of the related studies were cross-sectional in nature which could not establish the direction of the effects involved. To our best knowledge, there are three related longitudinal studies to date. Specifically, Yu and Shek (2013) reported that better family functioning characterized by better family mutuality, less conflicts and more communication among family members predicted a lower level of Chinese adolescents' IA 2 years later with the marginally significant effect (i.e., *p* = 0.06). However, in another study, the longitudinal predicting effect of family functioning on Chinese adolescents' IA in 3 years was not significant (Shek et al., 2015). For another exception, Ko et al. (2015) assessed IA and family factors among 2,293 Grade 7 students in Taiwan with a 1-year follow up. They found that conflict among parents and parents' insufficient regulation of children's unessential usage of the Internet significantly predicted the higher incidence of adolescent IA 1 year later. While these studies provided indirect or direct support for the longitudinal impacts of parenting, they did not distinguish between mothers' and fathers' influences with reference to different aspects of parent-child subsystem qualities.

In addition, there is an urgent need to conduct longitudinal studies to underscore whether and how parental factors may predict the rate of change in adolescent IA. A few studies found that adolescent IA may decrease over time during secondary school years (Yu and Shek, 2013; Shek et al., 2015; Shek and Yu, 2016). Although Shek et al. (2015) reported that adolescents in non-intact families or without economic disadvantages decreased their IA at a faster rate, the study did not consider different parenting factors as potential predictors. Theoretically, one can conjecture that better parent-child subsystem qualities can help adolescents cope with life stress encountered offline in a more constructive way and direct their efforts toward meaningful activities. Thus, positive parenting may be associated with adolescents' long-term positive adjustment such as faster decrease in IA. Therefore, utilizing longitudinal design to empirically investigate how parents' control and the quality of parent-child relationships are associated with the change in adolescent IA is necessary.

To fill the above-mentioned four research gaps, we utilized a large and representative sample of Chinese adolescents in Hong Kong to address the following three research question. First, we investigated whether behavioral control, psychological control, and quality of parent-child relationships of both parents predicted the initial level of adolescent IA. As previous literature suggested a negative linkage between parental behavioral control and adolescent IA (Lin et al., 2009; Bleakley et al., 2016; Ding et al., 2017) and a positive association between parental psychological control and adolescent IA (Giles and Price, 2008; Huang et al., 2010; Cheung et al., 2015), we hypothesized that both fathers' (Hypothesis 1a) and mothers' (Hypothesis 1b) behavioral control would negatively predict the initial level of adolescent IA, whereas both fathers' (Hypothesis 1c) and mothers' (Hypothesis 1d) psychological control would positively predict the initial level of adolescent IA. Furthermore, as a positive parent-child relationship would enhance behavioral control (Shek and Law, 2014) and adolescent IA may be a dysfunctional means of coping with unfavorable family relationships (Bleakley et al., 2016), we hypothesized that the quality of father-child relationship (Hypothesis 1e) and mother-child relationship (Hypothesis 1f) would negatively predict the initial level of adolescent IA.

Second, the present study explored how fathering and mothering with reference to different aspects of parent-child subsystem qualities predicted the rate of change in adolescent IA. Given that a better familial environment was associated with a faster drop in adolescent IA (Shek et al., 2015), we hypothesized that paternal behavioral control (Hypothesis 2a), maternal behavioral control (Hypothesis 2b), better father-child (Hypothesis 2c), and mother-child (Hypothesis 2d) relational qualities, as positive parenting characteristics, would predict a faster decrease in adolescent IA. In contrast, paternal (Hypothesis 2e) and maternal (Hypothesis 2f) psychological control, as negative parenting characteristics, would predict a slower drop in adolescent IA.

Finally, we explored relative contribution of different parenting characteristics (e.g., different mother-related factors and father-related factors) to the concurrent and future incidence of adolescent IA. Due to the inconclusive picture as to different influences of fathering and mothering on adolescent development and the limited evidence on the relative contribution of different parenting characteristics to adolescent IA, we did not generate specific hypotheses for this research question.

## Materials and methods

### Participants and procedures

In 2009/2010 academic year, a large-scale longitudinal project was launched in Hong Kong to investigate Chinese adolescents' adjustment and its antecedents. The project was approved by the Human Subjects Ethics Sub-committee (HSESC) (or its Delegate) of the authors' university. In total, 28 randomly selected secondary schools joined the project and all Grade 7 (i.e., Secondary 1) students in these schools were invited to respond to the same survey on a yearly basis during their secondary school lives. The survey consisted of multiple measures, including delinquency, IA, parent-child subsystem qualities (Shek and Ma, 2014; see Ma and Shek, 2017). Prior to the study, the participating schools and students' parents were well-informed and their written consent were obtained. Before each wave of data collection, the participating students were well explained the principles of voluntary participation and confidentiality and their written consent were also obtained.

The present study utilized data collected at Wave 1, 2, and 3, when students started their first, second, and third year of junior secondary school study, respectively. At Wave 1 data collection, 3,328 Grade 7 students (*M*_*age*_ = 12.59, *SD*_*age*_ = .74) responded to the survey, including 1,735 (52.1%) boys, 1,584 (47.6%) girls and, 9 (0.3%) who did not indicate their gender. Among these participants, 2,905 and 2,860 also completed the survey at the second and third wave of data collection, respectively, resulting in an acceptable attrition rate of 12.7% at Wave 2 and 14.1% at Wave 3. In all waves of data collection, the paper-and-pencil questionnaires were administrated by trained research staff in quiet classrooms in each participating school. The student respondents were clearly instructed to respond to each question in an honesty manner according to question instructions.

### Instruments

The questionnaire used in the survey was comprised of multiple measures, among which IA and parent-child subsystem qualities were the foci of the present paper. Besides, family economic status and family intactness as well as gender were statistically controlled in the current study.

#### Internet addiction (IA)

Young's 10-item IA Test has been translated into Chinese and validated by Shek et al. (2008). The present study measured adolescent IA with this Chinese version scale, which has been proved to have good psychometric properties and has been widely used in research involving Chinese adolescents (Shek et al., 2008; Shek and Yu, 2012a, 2016). The participants reported “Yes” or “No” to 10 statements to indicate whether they had showed the listed addicted behaviors related to the Internet during the last year. The total number of addicted behaviors the adolescents demonstrated was computed to index their IA. In this study, Cronbach's α of the IA Test ranged between 0.79 and 0.80 across waves (see Table 1).

**Table 1:** Reliability of scales and description of variables across the three waves.

**Scale**	**Number of item**	**Wave**	**Cronbach's α**	**Mean inter-item correlation**	**Range**	***M***	***SD***
Internet addiction test	10	Wave 1	0.79	0.28	0–10	2.22	2.32
		Wave 2	0.79	0.28	0–10	2.32	2.36
		Wave 3	0.80	0.29	0–10	2.04	2.27
Father-child subsystem quality scale	17						
Paternal behavioral control	7	Wave 1	0.89	0.54	1–4	2.56	0.67
		Wave 2	0.89	0.54	1–4	2.53	0.64
		Wave 3	0.89	0.53	1–4	2.50	0.63
Paternal psychological control	4	Wave 1	0.80	0.50	1–4	2.24	0.72
		Wave 2	0.83	0.54	1–4	2.26	0.72
		Wave 3	0.86	0.61	1–4	2.22	0.74
Father-child relational quality	6	Wave 1	0.90	0.60	1–4	2.80	0.70
		Wave 2	0.91	0.62	1–4	2.76	0.69
		Wave 3	0.90	0.62	1–4	2.75	0.67
Mother-child subsystem quality scale	17						
Maternal behavioral control	7	Wave 1	0.90	0.55	1–4	3.03	0.62
		Wave 2	0.89	0.54	1–4	2.96	0.60
		Wave 3	0.89	0.54	1–4	2.91	0.58
Maternal psychological control	4	Wave 1	0.85	0.59	1–4	2.31	0.77
		Wave 2	0.88	0.64	1–4	2.31	0.76
		Wave 3	0.89	0.67	1–4	2.27	0.76
Mother-child relational quality	6	Wave 1	0.91	0.63	1–4	3.05	0.67
		Wave 2	0.91	0.64	1–4	2.96	0.66
		Wave 3	0.90	0.62	1–4	2.96	0.62

#### Father- and mother-child subsystem qualities

The Parent-Child Subsystem Quality Scale (PCSQS), which possessed good psychometric properties (Shek and Law, 2014, 2015a), was used to measure father- and mother-child subsystem qualities. Quality of each subsystem was measured with the corresponding 17-item subscale pertinent to three dimensions: (1) paternal/maternal behavioral control comprised of paternal/maternal knowledge, expectation, and monitoring (7 items in total, e.g., “My father/mother asked me about what I did after school,” “my father/mother expects me to have good behavior in school,” and “my father/mother actively understands my afterschool activities”); (2) paternal/maternal psychological control measured by four items with one sample item as “Father/mother often wants to change my mind or feelings for things”; and (3) father-/mother-child relational quality indicated by the extent to which the participant satisfied with paternal/maternal control and the participant's active communication with his/her father/mother (six items in total, e.g., “my father's/mother's discipline of me is reasonable” and “I shared my feelings with my father/mother”). Participants reported how they agreed with each statement on a 4-point Likert scale (1 = “strongly disagree,” 4 = “strongly agree”). The average score in each dimension was calculated to indicate the corresponding process of parent-child subsystem qualities. As shown in Table 2, all subscales in the PCSQS showed good internal consistency with all Cronbach's α's ranging between 0.80 and 0.91 across waves.

**Table 2:** Correlations among variables.

**Variables**		**1**	**2**	**3**	**4**	**5**	**6**	**7**	**8**	**9**	**10**	**11**
1.	Gender^a^	−										
2.	FES^b^	0.03	−									
3.	FI^c^	0.005	0.35^***^	−								
4.	W1 PBC	0.03	0.14^***^	0.18^***^	−							
5.	W1 PPC	0.13^***^	0.03	0.03	0.20^***^	−						
6.	W1 FCRQ	−0.01	0.15^***^	0.21^***^	0.68^***^	−0.05^**^	−					
7.	W1 MBC	−0.06^***^	0.05^*^	0.11^***^	0.43^***^	0.07^***^	0.36^***^	−				
8.	W1 MPC	0.07^***^	−0.02	−0.04^*^	0.02	0.48^***^	−0.08^***^	0.11^***^	−			
9.	W1 MCRQ	−0.04^*^	0.04^*^	0.10^***^	0.38^***^	−0.004	0.46^***^	0.68^***^	−0.13^***^	−		
10.	W1 IA	0.05^**^	−0.03	−0.08^***^	−0.24^***^	0.10^***^	−0.27^***^	−0.16^***^	0.15^***^	−0.24^***^	−	
11.	W2 IA	0.04^*^	−0.01	−0.02	−0.12^***^	0.10^***^	−0.15^***^	−0.10^***^	0.12^***^	−0.13^***^	0.53^***^	−
12.	W3 IA	0.06^**^	−0.04^*^	0.003	−0.08^***^	0.09^***^	−0.11^***^	−0.08^***^	0.07^**^	−0.10^***^	0.42^***^	0.55^***^

*The correlational patterns between parent-child subsystem qualities at different waves and other variables were the same, so only the results on Wave 1 parenting characteristics were presented in the table due to space limit. FES, Family economic status; FI, Family intactness; PBC, Paternal behavioral control; PPC, Paternal psychological control; FCRQ, Father-child relational quality; MBC, Maternal behavioral control; MPC, Maternal psychological control; MCRQ, Mother-child relational quality; IA, Internet addiction; W1, Wave 1; W2, Wave 2; W3, Wave 3*.

a *Female = −1, Male = 1*.

b *Having economic disadvantage = −1, Not having economic disadvantage = 1*.

c *Non-intact = −1, Intact = 1*.

* p < 0.05;

** p < 0.01;

*** *p < 0.001*.

#### Family economic status

In Hong Kong, living on welfare of “Comprehensive Social Security Assistance (CSSA) Scheme” provided by the government is usually used to indicate family economic disadvantage. In the present study, 225 (6.8%) students had family economic disadvantage as they reported living on CSSA at Wave 1 and 2,606 (78.3%) respondents did not have family economic disadvantage as they were not living on CSSA.

#### Family intactness

Family intactness was operationalized as parents' marital status at Wave 1. A total of 2,781 (83.6%) adolescents whose parents were in the first marriage were regarded as living in intact family. Other 515 (15.5%) participants who reported their parents were separated, divorced, or in second marriage were regarded as having non-intact family.

### Attrition analysis

Across the three waves, 2,669 participants can be successfully matched (i.e., matched group) and 659 participants dropped out after Wave 1 (i.e., dropouts group). These two groups were compared with reference to socio-demographic profile and baseline condition of IA and parent-child subsystem qualities at Wave 1. Compared with the dropouts, the matched group was slightly younger [*t*_(3283)_ = −4.28, *p* < 0.001, Cohen's *d* = 0.20], included a larger proportion of girls [$\chi_{\left(\text{1}\right)}^{\text{2}}$ = 39.70, *p* < 0.001, ϕ = 0.11] and had more participants without family economic disadvantage [$\chi_{\left(\text{1}\right)}^{\text{2}}$ = 7.10, *p* = 0.01, ϕ = 0.05] or living in intact family [$\chi_{\left(\text{1}\right)}^{\text{2}}$ = 8.68, *p* = 0.004, ϕ = 0.05]. Further analyses showed that the matched group had a lower level of IA [*t*_(3324)_ = −4.22, *p* < 0.001, Cohen's *d* = 0.04], a higher level of maternal behavioral control, lower levels of fathers' and mothers' psychological controls, and better father- and mother-child relational qualities as compared to the dropouts (absolute *t*-value ranged between 2.15 and 5.74, *p*s < 0.05, Cohen's *d* ranged between 0.09 and 0.26).

Above results suggested a systematic attrition of certain participants, which might cause bias to the findings of the present study. To deal with this issue, we imputed the missing values in IA and parent-child subsystem qualities by adopting the procedure outlined in previous literature (Asendorpf et al., 2014). Specifically, we employed “Predictive Mean Matching” option via using “multiple imputation” approach incorporated in SPSS. In the current study, 40 times of imputation were performed, resulting in 41 data sets including the original one and the 40 sets of imputed data.

Statistical analyses including correlational analyses, individual growth curve (IGC) analyses, and multiple regression analyses were conducted based on each of the 41 data sets. For each statistical parameter, the average of corresponding values across the 40 sets of imputed data were computed as the pooled result (Rubin, 2004). Comparisons between the pooled results and those based on the original data yielded similar findings, indicating that the attrition did not lead to significant bias in the present study. Thus, we reported pooled results. The procedure utilized in the present study is recommended and widely adopted recently by researchers to examine and deal with attrition issue in longitudinal research (e.g., Asendorpf et al., 2014; Huijbers et al., 2015).

### Data analytic plan

Reliability, descriptive, and correlational analyses were performed first. To address the first and second research questions, i.e., to examine whether and how different aspects of parent-child subsystem qualities contribute to the initial level and the rate of change in adolescent IA, we employed the IGC analytic approach, which has been widely utilized to investigate the developmental course as well as its influencing factors at the individual level (Shek and Yu, 2012b; Shek and Lin, 2016).

Following the procedure used in previous studies (Shek and Ma, 2011; Shek and Yu, 2012b; Shek and Lin, 2016), we nested time as the level-1 predictor into individual characteristics as the level-2 predictors, resulting in several sets of 2-level hierarchical models. The test of each set of hierarchical models involved two steps. First, an unconditional mean model (Model 1) and a linear growth model (Model 2) that merely used level-1 predictors were tested to elucidate the overall developmental trajectory of IA. Time was coded as follows: Wave 1 = 0, Wave 2 = 1, and Wave 3 = 2. Second, control variables (Model 3) and different aspects of parent-child subsystem qualities (Models 4a−4c) measured at Wave 1 were examined as time-invariant covariates to explore whether these factors caused any individual variability in the growth curve of IA. Noteworthy, separate IGC analyses were performed for the three different dimensions of parent-child subsystem qualities.

To index model fit for each analysis, we used three indices: −2log likelihood, AIC (Akaike Information Criterion), and BIC (Bayesian Information Criterion). For all three indices, a small value represents a better model fit (Shek and Ma, 2011). Furthermore, to facilitate the IGC analyses, we dummy coded the three socio-demographic characteristics: male = 1, female = −1; not having economic disadvantage = 1, having economic disadvantage = −1; intact family = 1, non-intact family = −1. Meanwhile, different processes of parent-child subsystem qualities as the intended level-2 predictors were standardized so that in the level-2 models, the coefficients of each predictor in intercept and linear slope represented the changes in mean value and the linear growth slope of IA, respectively, per unit of change in the corresponding predictor.

To address the third research question mentioned before, we further conducted multiple regression analyses to examine and compare the predicting effects of different paternal factors, maternal factors, and all the related parenting factors, respectively, on the concurrent and future incidences of adolescent IA. As the cross-sectional effects at Wave 1 have been reported elsewhere (Shek and Law, 2015b), the present study looked at the cross-sectional effects at Wave 2 and Wave 3. To examine longitudinal effects, parenting factors at Wave 1 were used as the intended predictors and adolescent IA at Wave 2 and Wave 3 were used as the two dependent variables.

## Results

### Correlations among variables

The correlations among the considered variables are depicted in Table 2. While parents' psychological control had a positive correlation with adolescent IA, parents' behavioral control and the quality of parent-child relationships were negatively correlated with adolescent IA. The correlations also suggested that girls tended to have a lower level of IA than boys.

### Predicting effects on the initial level and the rate of change in adolescent IA

The results of the IGC analyses for the unconditional models are demonstrated in Table 3. First, the results of the unconditional mean model (i.e., Model 1) indicated an intra-class correlation coefficient (ICC) of 0.505, implying that 50.5% of the variance of IA was attributed to inter-personal differences. Therefore, there is a need to conduct multi-level analyses involving both level-1 and level-2 predictors (Shek and Ma, 2011). Moreover, the unconditional linear model (i.e., Model 2) fitted the data better than did Model 1 [Δ$\chi_{\left(\text{3}\right)}^{2}$ = 70.39; *p* < 0.001; ΔAIC = 64.39; ΔBIC = 43.43]. Based on Model 2, there was a significant negative linear slope in adolescent IA (β = −0.092, *p* < 0.001), meaning that adolescent IA slightly decreased during the junior secondary school years.

**Table 3:** Results of IGC models with level-1 predictors for adolescent Internet addiction (Waves 1–3).

			**Model 1**		**Model 2**	
			**Estimate**	**SE**	**Estimate**	**SE**
**FIXED EFFECTS**						
Intercept		*β_*0j*_*				
	Intercept	*γ_*00*_*	2.196^***^	0.0366	2.288^***^	0.0440
Linear Slope		*β_*1j*_*				
	Time	*γ_*10*_*			−0.092^***^	0.0240
**RANDOM EFFECTS**						
Level 1 (within)						
	Residual	*r_**i*j*_*	2.7135^***^	0.0525	2.3249^***^	0.0636
Level 2 (between)						
	Intercept	*u_*0j*_*	2.6650^***^	0.0993	3.2325^***^	0.1511
	Time	*u_*1j*_*			0.3801^***^	0.0529
**FIT STATISTICS**						
	Deviance		34379.69		34309.30	
	AIC		34385.69		34321.30	
	BIC		34406.66		34363.23	
	Df		3		6	

*Model 1, unconditional mean model; Model 2, unconditional linear growth model; AIC, Akaike Information Criterion; BIC, Bayesian Information Criterion*.

*** *p < 0.001*.

As the variability was significant in the intercept and linear slope in Model 2 (see Table 3), we further investigated the predictive effects of individual characteristics on the intercept and linear slope. As shown in Model 3 (see Tables 4–**6**), among the three control variables, only family intactness demonstrated significant predicting effects on the initial level and the rate of change in IA. Specifically, participants living in non-intact family showed a higher initial level (β = −0.209, *p* < 0.01) but a faster drop (β = 0.130, *p* < 0.001) in IA. After parent-child subsystem qualities were further considered, the predicting effect of family intactness on the rate of change in IA remained significant (see Tables 4–**6**).

**Table 4:** Results of IGC models with level-2 predictors for adolescent Internet addiction (Waves 1-3, Linear).

		**Model 3**		**Model 4a**	
		**Estimate**	**SE**	**Estimate**	**SE**
**FIXED EFFECTS**					
Intercept	β_*0j*_				
Intercept	γ_*00*_	2.459^***^	0.0840	2.327^***^	0.0826
Gender^a^	γ_*01*_	0.066	0.0442	0.073	0.0432
Family economic status^b^	γ_*02*_	−0.032	0.0846	0.038	0.0823
Family intactness^c^	γ*03*	−0.209^**^	0.0657	−0.100	0.0647
Paternal behavioral control	γ_*04*_			−0.436^***^	0.0482
Maternal behavioral control	γ_*05*_			−0.160^**^	0.0479
Linear Slope	*β_*1j*_*				
Intercept	γ_*10*_	−0.153^**^	0.0458	−0.107^*^	0.0457
Gender^a^	γ_*11*_	0.023	0.0241	0.018	0.0239
Family economic status^b^	γ_*12*_	−0.037	0.0459	−0.062	0.0456
Family intactness^c^	γ_*13*_	0.130^***^	0.0358	0.093^**^	0.0358
Paternal behavioral control	γ_*14*_			0.173^***^	0.0267
Maternal behavioral control	*dγ*_*15*_			0.013	0.0265
**RANDOM EFFECTS**					
Level 1 (within)					
Residual	*r_**ij**_*	2.3286^***^	0.0643	2.3285^***^	0.0643
Level 2 (between)					
Intercept	*u_*0*j**_*	3.1820^***^	0.1513	2.9181^***^	0.1445
Time	*u_*1j*_*	0.3553^***^	0.0529	0.3245^***^	0.0522
**FIT STATISTICS**					
Deviance		33645.70		33502.77	
AIC		33669.70		33534.77	
BIC		33753.34		33646.29	
df		12		16	

*Model 3, conditional growth curve model (only with socio-demographic variables); Model 4a, conditional growth curve model (adding parental behavioral control). AIC, Akaike Information Criterion; BIC, Bayesian Information Criterion*.

a *Female = −1, Male = 1*.

b *Having economic disadvantage = −1, Not having economic disadvantage = 1*.

c *Non-intact = −1, Intact = 1*.

* *p < 0.05*.

** *p < 0.01*.

*** *p < 0.001*.

As demonstrated in Table 4, compared to Model 3 which only included the control variables, Model 4a which included paternal and maternal behavioral control had a better model fit [Δ$\chi_{\left(4\right)}^{\text{2}}$ = 142.93; *p* < 0.001; ΔAIC = 134.93; ΔBIC = 107.05]. Model 4a also fitted the data better than did Model 2 [Δ$\chi_{\left(10\right)}^{\text{2}}$ = 806.53; *p* < 0.001; ΔAIC = 777.53; ΔBIC = 716.94]. Likewise, both Model 4b which involved control variables and parental psychological control (see Table 5) and Model 4c that considered control variables and parent-child relational qualities (see Table 6) had a better model fit as compared to Model 2 and Model 3. Therefore, the results were interpreted based on Models 4a, 4b, and 4c.

**Table 5:** Results of IGC models with level-2 predictors for adolescent Internet addiction (Waves 1–3, Linear).

		**Model 3**		**Model 4b**	
		**Estimate**	**SE**	**Estimate**	**SE**
**FIXED EFFECTS**					
Intercept	*β_*0j*_*				
Intercept	γ_00_	2.459^***^	0.0840	2.449^***^	0.0832
Gender^a^	γ_01_	0.066	0.0442	0.031	0.0441
Family economic status^b^	γ_02_	−0.032	0.0846	−0.034	0.0834
Family intactness^c^	γ_03_	−0.209^**^	0.0657	−0.193^**^	0.0651
Paternal psychological control	γ_04_			0.117^*^	0.0498
Maternal psychological control	γ_05_			0.279^***^	0.0495
Linear slope	*β_*1*j**_*				
Intercept	γ_10_	−0.153^**^	0.0458	−0.147^**^	0.0457
Gender^a^	γ_11_	0.023	0.0241	0.029	0.0242
Family economic status^b^	γ_12_	−0.037	0.0459	−0.039	0.0458
Family intactness^c^	γ_13_	0.130^***^	0.0358	0.124^*^	0.0358
Paternal psychological control	γ_14_			0.010	0.0274
Maternal psychological control	γ_15_			−0.095^**^	0.0272
**RANDOM EFFECTS**					
Level 1 (within)					
Residual	*r_*ij*_*	2.3286^***^	0.0643	2.3286^***^	0.0643
Level 2 (between)					
Intercept	*u_0*j*_*	3.1820^***^	0.1513	3.0610^***^	0.1482
Time	*u_1*j*_*	0.3553^***^	0.0529	0.3471^***^	0.0527
**FIT STATISTICS**					
Deviance		33645.70		33578.79	
AIC		33669.70		33610.79	
BIC		33753.34		33722.31	
df		12		16	

*Model 3, conditional growth curve model (only with socio-demographic variables); Model 4b, conditional growth curve model (adding parental psychological control). AIC, Akaike Information Criterion; BIC, Bayesian Information Criterion*.

a Female = −1, Male = 1;

b *Having economic disadvantage = −1, Not having economic disadvantage = 1*.

c *Non-intact = −1, Intact = 1*.

* *p < 0.05*.

** *p < 0.01*.

*** *p < 0.001*.

**Table 6:** Results of IGC models with level-2 predictors for adolescent Internet addiction (Waves 1–3, Linear).

		**Model 3**		**Model 4c**	
		**Estimate**	**SE**	**Estimate**	**SE**
**FIXED EFFECTS**					
Intercept	β_*0j*_				
Intercept	γ_*00*_	2.459^***^	0.0840	2.297^***^	0.0818
Gender^a^	γ_*01*_	0.066	0.0442	0.057	0.0425
Family economic status^b^	γ_*02*_	−0.032	0.0846	0.044	0.0813
Family intactness^c^	γ_*03*_	−0.209^**^	0.0657	−0.063	0.0641
Father-child relational quality	γ_*04*_			−0.421^***^	0.0484
Mother-child relational quality	γ_*05*_			−0.335^***^	.0477
Linear slope	β_*1j*_				
Intercept	γ_*10*_	−0.153^**^	0.0458	−0.104^*^	0.0458
Gender^a^	γ_*11*_	0.023	0.0241	0.025	0.0238
Family economic status^b^	γ_*12*_	−0.037	0.0459	−0.061	0.0456
Family intactness^c^	γ_*13*_	0.130^***^	0.0358	0.085^*^	0.0359
Father-child relational quality	γ_*14*_			0.135^***^	0.0272
Mother-child relational quality	γ_*15*_			0.087^**^	0.0267
**RANDOM EFFECTS**					
Level 1 (within)					
Residual	*r_**ij**_*	2.3286^***^	0.0643	2.3286^***^	0.0643
Level 2 (between)					
Intercept	*u_*0*j**_*	3.1820^***^	0.1513	2.7865^***^	0.1412
Time	*u_*1*j**_*	0.3553^***^	0.0529	0.3207^***^	0.0521
**FIT STATISTICS**					
Deviance		33645.70		33434.69	
AIC		33669.70		33466.69	
BIC		33753.34		33578.21	
df		12		16	

*Model 3, conditional growth curve model (only with socio-demographic variables); Model 4c, conditional growth curve model (adding parent-child relational qualities). AIC, Akaike Information Criterion; BIC, Bayesian Information Criterion*.

a *Female = −1, Male = 1*.

b *Having economic disadvantage = −1, Not having economic disadvantage = 1*.

c *Non-intact = −1, Intact = 1*.

* *p < 0.05*.

** *p < 0.01*.

*** *p < 0.001*.

**Figure 1:**
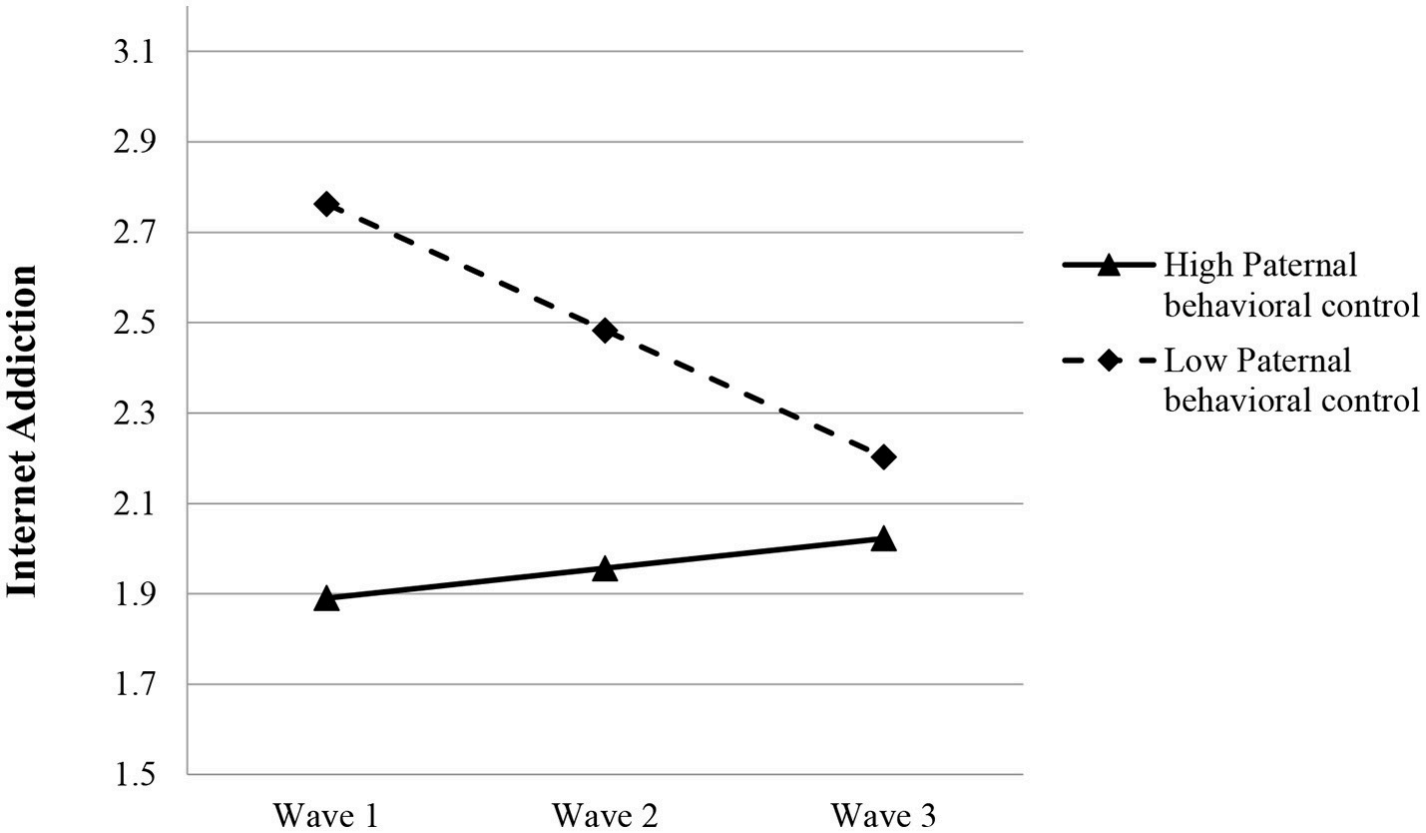
Growth trajectories of adolescent internet addiction as a function of paternal behavioral control. The figures were plotted based on Model 4a shown in Table 4. High level indicates 1SD higher than the mean value; low level indicates 1SD lower than the mean value.

Based on Model 4a, while both paternal (β = −0.436, *p* < 0.001) and maternal behavioral control (β = −0.160, *p* < 0.01) showed negative predicting effects on the initial level of adolescent IA, only paternal behavioral control showed a significant positive relationship with the rate of linear change in IA (β = 0.173, *p* < 0.001) (see Model 4a in Table 4), suggesting that higher paternal behavioral control predicted a slower drop in IA over time (see Figure 1).

**Figure 2:**
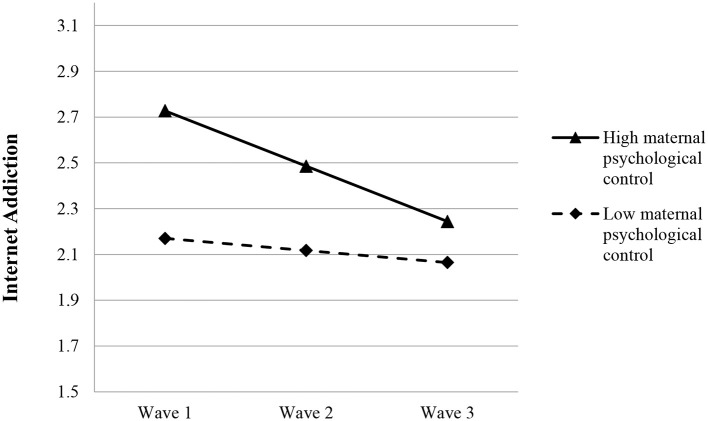
Growth trajectories of adolescent Internet addiction as a function of maternal psychological control. The figures were plotted based on Model 4b shown in Table 5. High level indicates 1SD higher than the mean value; low level indicates 1SD lower than the mean value.

Based on Model 4b, while both paternal (β = 0.117, *p* < 0.05) and maternal psychological control (β = 0.279, *p* < 0.001) were positively related to the initial level of adolescent IA, only maternal psychological control showed a significant negative predicting effect on the rate of change in IA (β = −0.095, *p* < 0.01) (see Model 4b in Table 5), indicating that higher maternal psychological control predicted a faster drop in adolescent IA (see Figure 2).

**Figure 3:**
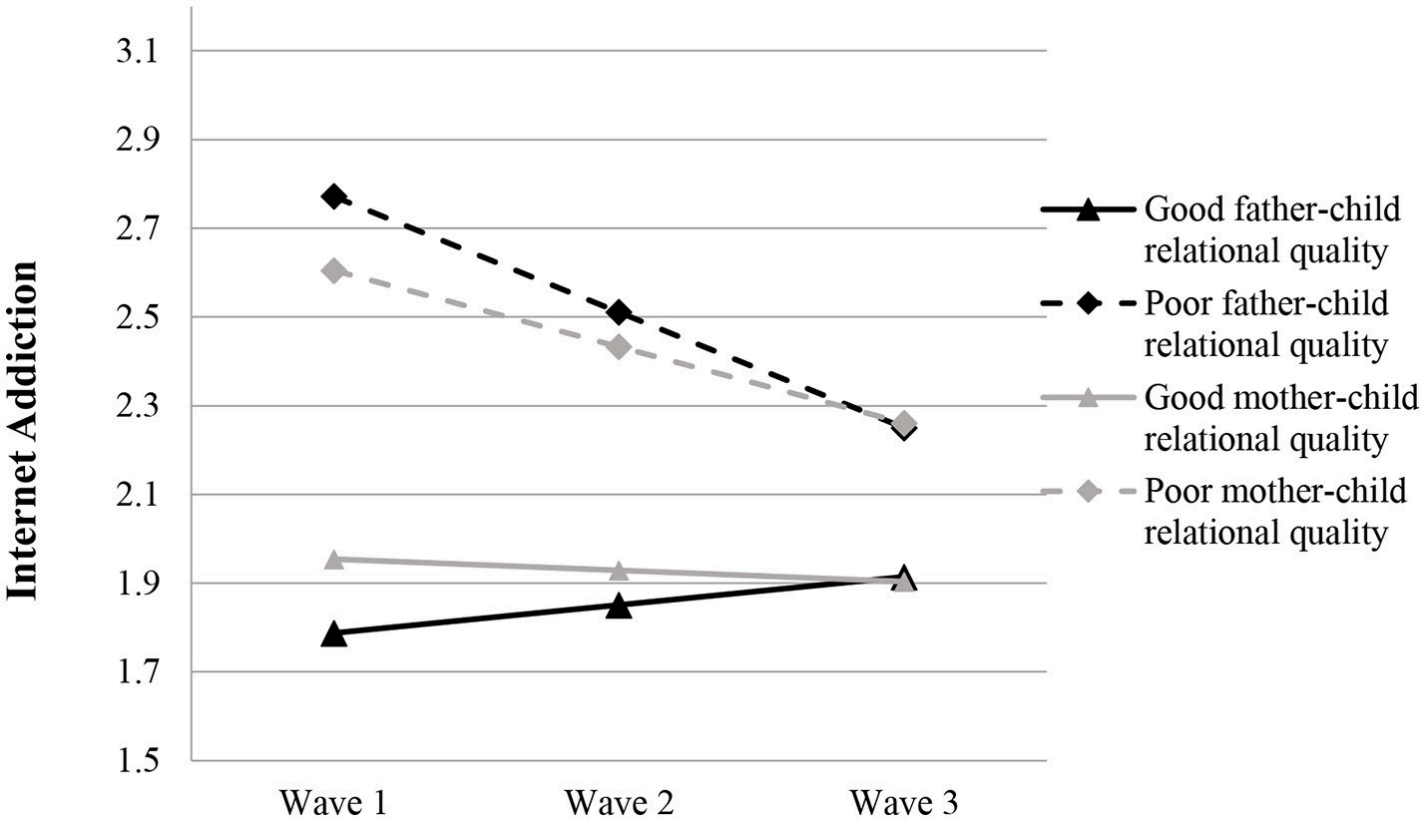
Growth trajectories of adolescent Internet addiction as a function of parent-child relational qualities. The figures were plotted based on Model 4c shown in Table 6. Good quality indicates 1SD higher than the mean value; poor quality indicates 1SD lower than the mean value.

Based on Model 4c, father-child relational quality (β = −0.421, *p* < 0.001) and mother-child relational quality (β = −0.335, *p* < 0.001) negatively predicted the initial level of IA but positively predicted the change rate of IA (father: β = 0.135, *p* < 0.001; mother: β = 0.087, *p* < 0.01) (see Model 4c in Table 6), suggesting that poorer parent-child relational qualities were associated with a faster drop in adolescent IA (see Figure 3).

### Relative contribution of paternal and maternal factors

To address the third research question, multiple regression analyses regarding the cross-sectional and longitudinal effects of parenting characteristics were performed. Results are shown in Table 7.

**Table 7:** Concurrent and longitudinal predicting effects of parent-child subsystem qualities on Internet addiction.

**Model**	**Predictors**	**Concurrent predicting effects**						**Longitudinal predicting effects**					
		**Wave 2 Internet addiction^a^**			**Wave 3 Internet addiction^b^**			**Wave 2 Internet addiction^c^**			**Wave 3 Internet addiction^c^**		
		**β**	***t***	**Cohen's *f^2^***	**β**	***t***	**Cohen's *f^2^***	**β**	***t***	**Cohen's *f^2^***	**β**	***T***	**Cohen's *f^2^***
1	Gender^d^	0.04	1.79^∧^	0.001	0.05	2.63^**^	0.003	0.04	1.79^∧^	0.001	0.05	2.63^**^	0.003
	FES^e^	0.01	0.22	0.000	−0.04	−1.78^∧^	0.001	0.01	0.22	0.000	−0.04	−1.78^∧^	0.001
	FI^f^	−0.02	−1.08	0.000	0.02	0.73	0.000	−0.02	−1.08	0.000	0.02	0.73	0.000
	*R^2^* change	0.002			0.004			0.002			0.004		
	*F* change	1.451			3.29^*^			1.451			3.29^*^		
2	PBC	−0.12	−4.46^***^	0.008	−0.08	−3.00^**^	0.003	−0.08	−3.03^**^	0.003	−0.03	−1.18	0.001
	PPC	0.11	5.17^***^	0.010	0.15	7.29^***^	0.020	0.10	4.98^***^	0.009	0.08	3.67^***^	0.005
	FCRQ	−0.05	−1.89^∧^	0.001	−0.04	−1.36	0.001	−0.08	−2.75^**^	0.003	−0.08	−3.02^**^	0.003
	*R^2^* change	0.035			0.034			0.029			0.017		
	*F* change	31.23^***^			30.85^***^			25.91^***^			15.04^***^		
3	MBC	−0.01	−0.50	0.000	−0.03	−1.18	0.001	−0.04	−1.56	0.001	−0.04	−1.35	0.001
	MPC	0.12	5.85^***^	0.013	0.15	6.95^***^	0.019	0.10	4.77^***^	0.009	0.06	2.79^**^	0.003
	MCRQ	−0.13	−4.72^***^	0.009	−0.06	−2.11^*^	0.002	−0.09	−3.37^**^	0.004	−0.08	−2.81^**^	0.003
	*R^2^* change	0.040			0.032			0.026			0.015		
	*F* change	36.75^***^			28.89^***^			23.61^***^			13.27^***^		
4	PBC	−0.11	−4.08^***^	0.006	−0.07	−2.53^**^	0.002	−0.06	−2.21^*^	0.002	−0.02	−0.51	0.000
	PPC	0.06	2.68^**^	0.003	0.11	4.53^***^	0.008	0.07	3.21^**^	0.004	0.07	2.81^**^	0.003
	FCRQ	−0.01	−0.41	0.000	−0.02	−0.61	0.000	−0.05	−1.71^∧^	0.001	−0.06	−2.07^*^	0.002
	MBC	0.03	1.19	0.001	0.002	0.08	0.000	−0.02	−0.60	0.000	−0.03	−0.90	0.000
	MPC	0.09	3.88^***^	0.006	0.09	3.94^***^	0.006	0.06	2.59^*^	0.003	0.02	1.02	0.000
	MCRQ	−0.11	−4.08^***^	0.006	−0.05	−1.85^∧^	0.001	−0.07	−2.48^*^	0.002	−0.06	−2.04^*^	0.002
	*R^2^* change	0.054			0.045			0.038			0.022		
	*F* change	24.67^***^			20.74^***^			17.12^***^			9.95^***^		

*For models 2–4, social-demographic variables were controlled. FES, Family economic status; FI, Family intactness; PBC, Paternal behavioral control; PPC, Paternal psychological control; FCRQ, Father-child relational quality; MBC, Maternal behavioral control; MPC, Maternal psychological control; MCRQ, Mather-child relational quality*.

a *Parent-child subsystem qualities measured at Wave 2 were used*.

b *Parent-child subsystem qualities measured at Wave 3 were used*.

c *Parent-child subsystem qualities measured at Wave 1 were used*.

d *Female = 1, Male = 1*.

e *Having economic disadvantage = −1, Not having economic disadvantage = 1*.

f *Non-intact = −1, Intact = 1*.

∧ *p < 0.10*.

* *p < 0.05*.

** *p < 0.01*.

*** *p < 0.001*.

First, father-related factors measured at corresponding occasions were entered in the regression models as the intended predictors. As indicated in Table 7, three concurrent father-related factors uniquely accounted for 3.5 and 3.4% of the variance in adolescent IA at Wave 2 and Wave 3, respectively. To illustrate, while father-child relational quality only showed a marginal cross-sectional effect at Wave 2 (β = −0.05, *p* < 0.10, Cohen's *f* ^2^ = 0.001), paternal behavioral control was a negative concurrent predictor at Wave 2 (β = −0.12, *p* < 0.001, Cohen's *f* ^2^ = 0.008) and Wave 3 (β = −0.08, *p* < 0.01, Cohen's *f* ^2^ = 0.003), and psychological control was a positive concurrent predictor at Wave 2 (β = 0.11, *p* < 0.001, Cohen's *f* ^2^ = 0.010) and Wave 3 (β = 0.15, *p* < 0.001, Cohen's *f* ^2^ = 0.020). Regarding the longitudinal effect, three father-related factors measured at Wave 1 uniquely accounted for 2.9 and 1.7% of the variance in adolescent IA at Wave 2 and Wave 3, respectively. As shown in Table 7, paternal behavioral control at Wave 1 served as a negative predictor of adolescent IA at Wave 2 (β = −0.08, *p* < 0.01, Cohen's *f*^2^ = 0.003), but not at Wave 3 (β = −0.03, *p* > 0.05). In contrast, higher paternal psychological control at Wave 1 predicted higher IA at Wave 2 (β = 0.10, *p* < 0.001, Cohen's *f*^2^ = *0*.009) and Wave 3 (β = 0.08, *p* < 0.001, Cohen's *f* ^2^ = *0*.005) while better father-child relational quality at Wave 1 predicted lower IA at Wave 2 (β = −0.08, *p* < 0.01, Cohen's *f* ^2^ = 0.003) and Wave 3 (β = −0.08, *p* < 0.01, Cohen's *f* ^2^ = 0.003).

Second, similar procedures of multiple regression analysis were applied to address the relative contribution of different maternal factors. It was found that concurrent maternal factors uniquely explained 4.0 and 3.2% of the variance in adolescent IA at Wave 2 and Wave 3, respectively. As shown in Table 7, while maternal behavioral control did not have significant concurrent predicting effects on adolescent IA, mothers' psychological control exerted positive cross-sectional effects at Wave 2 (β = 0.12, *p* < 0.001, Cohen's *f* ^2^ = 0.013) and Wave 3 (β = 0.15, *p* < 0.001, Cohen's *f* ^2^ = 0.019), and the quality of mother-child relationship was a negative concurrent predictor at Wave 2 (β = −0.13, *p* < 0.001, Cohen's *f* ^2^ = 0.009) and Wave 3 (β = −0.06, *p* < 0.05, Cohen's *f* ^2^ = 0.002). With reference to longitudinal effects, mother-related factors at Wave 1 uniquely accounted for 2.6 and 1.5% of the variance in adolescent IA at Wave 2 and Wave 3, respectively. As can be seen in Table 7, mothers' behavioral control did not have significant longitudinal predicting effect on adolescent IA. In contrast, higher maternal psychological control at Wave 1 predicted higher incidence of IA at Wave 2 (β = 0.10, *p* < 0.001, Cohen's *f* ^2^ = 0.009) and Wave 3 (β = 0.06, *p* < 0.01, Cohen's *f* ^2^ = 0.003) whereas better mother-child relational quality at Wave 1 predicted lower IA at Wave 2 (β = −0.09, *p* < 0.01, Cohen's *f* ^2^ = 0.004) and Wave 3 (β = −0.08, *p* < 0.01, Cohen's *f* ^2^ = 0.003).

Finally, when all concurrent parenting characteristics were examined simultaneously, they uniquely accounted for 5.4 and 4.5% of the variance in IA at Wave 2 and Wave 3, respectively (see Table 7). Specifically, fathers' behavior control and psychological control as well as mothers' psychological control and mother-child relational quality were found to be the significant (or marginal significant) concurrent predictors at Wave 2 and Wave 3. As shown in Table 7, the parenting factors at Wave 1 uniquely explained 3.8 and 2.2% of the variance in adolescent IA at Wave 2 and Wave 3, respectively. Based on the results shown in Table 7, all three father-related factors and two mother-related factors (i.e., mothers' psychological control and the quality of mother-child relationship) were the significant (or marginal significant) longitudinal predictors of IA at Wave 2. For adolescent IA at Wave 3, paternal psychological control (β = 0.07 *p* < 0.01, Cohen's *f* ^2^ = 0.003), father-child relational quality (β = −0.06, *p* < 0.05, Cohen's *f* ^2^ = 0.002), and mother-child relational quality (β = −0.06, *p* < 0.05, Cohen's *f* ^2^ = 0.002) at Wave 1 were the significant predictors.

## Discussion

This study attempted to examine three research questions. First, we studied the predicting effects of paternal and maternal behavioral control and psychological control as well as quality of father- and mother-child relationships on the initial level in adolescent IA during the early adolescence. Second, we examined the influence of the parental control and parent-child relational quality measures on the rate of change in IA which is rarely examined in the literature. Third, the predicting effects of parenting characteristics on adolescent IA at different time points were also explored and compared. While the overall findings about the predicting effects on the levels of IA are in line with the extant theoretical propositions, findings on how parental factors predicted the rate of change in adolescent IA are novel. Generally speaking, the current findings add to the existing literature by underscoring the relative contribution of different processes of the parent-child subsystem qualities in influencing the level as well as the rate of change in adolescent IA and revealing how the parental impacts may change over time.

For the first research question, the findings supported Hypotheses 1a, 1b, 1c, 1d, 1e, and 1f. The present study found that both parents' behavioral control and the quality of father- and mother-child relationships were negatively associated with the initial level of adolescent IA, while parents' psychological control was positively related to the initial level of adolescent IA. These findings support the argument that positive parenting leads to better child outcomes (Barber et al., 2005; Shek, 2010). Specifically, behavioral control can be regarded as a protective factor for adolescent development, possibly by inhibiting their deviant behaviors such as problematic Internet use and facilitating adolescents to engage in other meaningful activities (Barber et al., 2005). Likewise, good parent-child relationships characterized by positive interaction (e.g., high-quality communication) are also beneficial to adolescents as the positive relationships lay a solid emotional foundation, which drives adolescents to behave in a desirable manner and thus prevents them from getting addicted to the Internet (Shek, 2010; Floros and Siomos, 2013). In contrast, psychological control which interferes with the fulfillment of the needs of autonomy and independence may reinforce the excessive usage of the Internet as a form of dysfunctional coping with stressful daily life events (Li et al., 2013; Wu et al., 2016).

For the second research question, it is noteworthy that the present study is among the pioneer attempts to explore how parenting characteristics predict the rate of change in adolescent IA using individual growth curving (IGC) analytical approach. However, the results are out of our expectations. First, only some of the parenting characteristics (i.e., fathers' behavioral control, mothers' psychological control and father- as well as mother-child relational qualities) were significantly related to the rate of change in adolescent IA. Second, the direction of the significant associations is at odds with the expectations. Specifically, lower behavioral control of fathers, higher psychological control of mothers, and poorer father- and mother-child relational qualities predicted a faster rate of decrease in adolescent IA. In short, Hypotheses 2a, 2b, 2c, 2d, 2e, and 2f were not supported.

The results may imply that parental impacts tend to diminish over time during early adolescence. At Wave 1, adolescents differed in their IA due to different parenting conditions with reference to the above mentioned four parenting characteristics. However, the divergence in adolescent IA attributable to parental impacts seemed to narrow gradually from Wave 1 to Wave 3. It is possible that parental influence may decrease as the child becomes more independent and turns to form important social networks outside of family such as peer relationships. In fact, some studies showed that peer-related (e.g., peer affiliation) and school-related factors (e.g., teacher-student relationship) also significantly contribute to adolescents' IA (Li et al., 2016; Jia et al., 2017).

Nevertheless, some of the parental effects (e.g., fathers' behavioral control and mothers' psychological control) seem to be more likely to decline over time than others (e.g., paternal psychological control). To some extent, these findings coincide with longitudinal predicting effects of different parenting characteristics derived from regression analyses, which will be discussed below. For instance, as the effects of fathers' behavioral control and mothers' psychological control decreased over time, these two constructs did not have significant longitudinal predictive effects on adolescent IA two years later. In this sense, results obtained from different analytical strategies are consistent with each other, which supports the reliability of these findings. While our results stress the importance of distinguishing between different processes of parenting as well as between fathering and mothering, it is necessary to conduct replication studies to verify the present novel findings and understand possible mechanisms involved.

The findings of the influence of parenting characteristics on the initial level of IA (i.e., the first research question) suggest that when each process of parent-child subsystem qualities was investigated separately, fathering and mothering tend to function similarly. However, findings of the third research question suggest that when different processes were examined simultaneously, fathering and mothering showed both similarities and differences in their concurrent and longitudinal predicting effects on the level of adolescent IA. Regarding the longitudinal parental effects on adolescent IA, limited evidence is available in the extant literature. Besides, the few exceptional studies only concerned with aggregated parenting with reference to one aspect such as behavioral control (Wang et al., 2013; Ko et al., 2015). In this sense, the present study goes beyond the existing scope by differentiating between fathering and mothering with regard to different processes of parent-child subsystem qualities.

For the third research question (i.e., relative contribution of father-related factors and mother-related factors), three related findings are highlighted below. First, fathers', but not mothers', behavioral control was a significant negative predictor of concurrent adolescent IA at Wave 2 and Wave 3. The result seems to contrast with the previous cross-sectional finding showing that mothers' rather than fathers' knowledge about children's activities (i.e., one dimension of behavioral control) was negatively related to adolescent deviance (Waizenhofer et al., 2004). However, this study did not consider other parenting characteristics and the involved sample size was relatively small (*N* = 95). In addition, the present study showed that paternal behavioral control at Wave 1 was negatively associated with later adolescent IA in one year. Such a longitudinal predicting effect diminished over time as it was not significant at Wave 3. On the contrary, maternal behavioral control at Wave 1 did not have significant longitudinal effect on adolescent IA. These results are in line with the finding in a recent longitudinal study (Lansford et al., 2014), which identified a unique predictive effect of paternal knowledge, but not maternal knowledge, on male adolescents' externalizing problems. The unique effect of paternal behavioral control was observed after socio-demographic variables and other parenting factors were statistically controlled. In addition, the present sample was large and representative. Therefore, we believe that our findings highlight the unique benefits ascribed to fathers' behavioral control.

Second, among the three father-related factors, psychological control appeared to be the strongest concurrent predictor at Wave 2 and Wave 3. Furthermore, higher paternal psychological control at Wave 1 significantly predicted higher adolescent IA at Wave 2 and Wave 3. Likewise, mothers' psychological control also showed significant concurrent and longitudinal positive predicting effects on IA, with the longitudinal effect declining over time. The present results not only corroborate general parenting literature showing the negative influence of psychological control on child development (Huang et al., 2010; Cheung et al., 2015), but also demonstrate that such negative influence could be long-lasting, especially for fathers' psychological control.

Regarding the cross-sectional effects, some studies found that mothers' psychological control served as a stronger predictor of children's problematic computer use than did fathers' psychological control (Giles and Price, 2008). Perhaps, mothers are more psychologically controlling than fathers, which may result in a stronger linkage between maternal psychological control and adolescent adjustment (Giles and Price, 2008). In contrast to this notion, our findings suggest that paternal and maternal psychological control may have similar cross-sectional effects on adolescent IA. One reason is that adolescents in the present study also perceived a considerable level of paternal psychological control. In fact, there are also studies which found that fathers' psychological control, as compared to mothers' psychological control, served as a more important concurrent predictor of adolescent internalizing and externalizing problems (Rogers et al., 2003). Despite these seemingly inconsistent findings, our results further suggest that fathers' psychological control may be as important as mothers' in early adolescence, or even more important when further concerning its longitudinal effect.

While the negative longitudinal effect of maternal psychological control declined over time, the negative influence of paternal psychological control remained robust across waves. Furthermore, when all the parenting factors were examined simultaneously, fathers', but not mothers', psychological control was positively associated with later adolescent IA in 2 years (i.e., at Wave 3). To some extent, our results echo the previous findings showing that high paternal psychological control was more likely to result in later externalizing problems in adolescents (Rogers et al., 2003; Lansford et al., 2014). Fathers' negative parenting in terms of psychological control might be especially detrimental due to its long-lasting negative impact. One possible explanation is that father's negative parenting is less likely to be counteracted by other positive relational factors of the father-child dyad, which is characterized by greater authority of the father and less closeness compared with the mother-child dyad (Rogers et al., 2003).

Third, while mother-child relational quality was significantly related to the concurrent adolescent IA at Wave 2 and Wave 3 with a stronger linkage at Wave 2, father-child relational quality was not a significant concurrent predictor at the two waves. However, father- and mother-child relational qualities at Wave 1 were the significant (or marginal significant) longitudinal predictors of later adolescent IA. On one hand, the results imply that the father- and mother-child relational qualities could exert over-time effects on adolescent IA throughout the early adolescence. This interpretation advocates the argument that relational quality lays a solid foundation for the impacts of other parenting factors (Crouter and Head, 2002). On the other hand, while the relational quality of mother-child dyad at different time point may be of similar importance, the relational quality of father-child dyad at an earlier stage might be more influential. In this sense, the particular importance of mother-child relational quality at the latter two time points echoes the finding in previous research, which showed that mother-child relationship had a stronger association with adolescent IA than did father-child relationship (Xu et al., 2014). Future studies are in need to replicate and extend the present findings by looking into different dimensions of parent-child relational qualities, such as mutual trust and communication.

The present study adds to the growing body of evidence that different processes in parent-child subsystem qualities are differentially related to adolescent IA, with reference to its concurrent and future incidence as well the rate of change during early adolescence. Nevertheless, several weaknesses of the study must be considered as well. First, as all considered variables were measured through adolescents' self-report, future studies will certainly benefit from utilizing multiple informants (e.g., parents and teachers) and different methods to collect data (e.g., diary report and interviews). In addition, due to repeated questionnaire administration, one may concern that students might give repeated responses or biased responses after learning the research objectives. Such a potential issue is a typical limitation for longitudinal psychological research. However, in the present study, adolescents were clearly instructed to give their honest responses based on the current status (parenting characteristics) or the situation in past 1-year (IA). We believe that students' responses would reflect their current perceptions instead of previous responses in memory. In addition, with the time period of 1 year, memory effect is even less likely to affect the results.

Second, although the findings obtained in this study could be regarded as having good generalizability for the adolescent population in Hong Kong, future research should involve adolescents from other geographic locations and ethnicity to enlarge the universality of the findings. Third, it is possible that certain dimension(s) of each process of parent-child subsystem qualities would have stronger influences on adolescent IA than others. For example, Li et al.'s (2013) cross-sectional study showed stronger associations between problematic Internet use and parental restriction as a form of behavioral control and love withdrawal as a form of psychological control. Future studies would benefit from further investigating longitudinal impacts of different dimensions of behavioral control, psychological control, and quality of parent-child relationships. Fourth, in the current study, we addressed related questions within early adolescent years. It will be illuminating if future research could investigate how different processes of parent-child subsystem qualities would influence the levels and the rate of change in adolescent IA over a longer period, such as the entire adolescent stage.

Finally, we used traditional in-class paper-and-pencil instead of computerized questionnaire. Although computer-based questionnaire administration merits such as perceived privacy and the usefulness in collecting sensitive information among adolescents (Dillman, 2000), it is quite demanding on participating schools' computer facilities. Besides, recent studies revealed that paper-and-pencil survey had a higher response rate and less missing data than did computerized one (Denniston et al., 2010; Wyrick and Bond, 2011). Taking these issues into consideration, the paper-and-pencil administration method is more operationally feasible for school-based survey and can be regarded as a good choice to ensure data quality of the present multi-year longitudinal study.

## Author contributions

DS designed the project and contributed to all steps of the work. XZ contributed to the data interpretation of the work, drafted the work, and revised it based on the critical comments provided by DS and CM. CM contributed to the refinement of the paper. All authors approve of the final version of the manuscript and agree to be accountable for all aspects of the work in ensuring that questions related to the accuracy or integrity of any part of the work are appropriately investigated and resolved.

### Conflict of interest statement

The authors declare that the research was conducted in the absence of any commercial or financial relationships that could be construed as a potential conflict of interest.
